# The International Medical Congress of 1887

**Published:** 1885-09

**Authors:** 


### EDITORIAL.

### THE INTERNATIONAL MEDICAL CONGRESS OF 1887.

The time appointed for the next meeting in New York, on September 3rd, of the Committee of Arrangements for *The Ninth International Medical Congress* is near at hand, and we desire to express the hope that the meeting may be characterized by evidence of appreciation of the responsibility of the position of that committee. The out-look for the congress is critical, and the honor of the medical profession of the United States is involved in the success of that congress. Therefore those who have been intrusted with the preparation for it should weigh well every official act of theirs. Too many mistakes have already been made. The present committee cannot afford to add to that number, any more than the profession at large can afford to have it done. Broad, liberal ground should be taken by the committee. It has the experience of the eight preceding congresses to guide it. Surely the experience gained in them should greatly aid the present committee, and the advantages of that experience should not be lost or ignored.

In the organization of the congress, the same rules should govern in the next that have obtained in the past. No distinction should be made between the foreign and the American members. The fact should be distinctly kept prominent that it is not a delegated body. It has not been in the past; there is no valid reason why there should be any effort to make the next one such. There should not be two or more classes of members. All must meet on the same plane—with equal privileges—equal duties—and equal responsibilities. Let all local ethical questions be eliminated. The foreign representatives expect and demand this, and such is their right. There may be honest differences of opinion, in our own country, on certain points of principle and policy, but there can be no valid reason for thrusting them, or their effects, upon foreigners who may come here. Sight should not be lost of the fact that science and humanity are the objects for which these congresses convene, and that all matters of selfishness and partisanship should be subordinated thereto.

As our faith, as a profession, was pledged, when our invitation was accepted, to do all in our power to make a success of the congress, we cannot do less. Everything that might conflict therewith, and diminish the prospect of such success, must be kept in abeyance.

For the official positions—whether the most prominent or the subordinate ones—only representative men should be selected. Much of the success or failure of the congress will depend upon the wisdom and the fairness of the action of the committee at its coming meeting. We hope that it will be such that there may be no occasion for vain regret.

